# The use of tirzepatide to successfully treat persistent genital arousal disorder/genitopelvic dysesthesia: a case report

**DOI:** 10.1093/sexmed/qfaf073

**Published:** 2025-09-24

**Authors:** Eliza Burr, Maya Roytman, Évéline Poirier, Marta Kolbuszewska, James G Pfaus, Barry R Komisaruk, Irwin Goldstein, Rachel Rubin

**Affiliations:** College of Human Medicine, Michigan State University, East Lansing, MI 48824, United States; Sexual Medicine Research Team, Washington, DC 20001, United States; Sexual Medicine Research Team, Washington, DC 20001, United States; Stritch School of Medicine, Loyola University Chicago, Chicago, IL 60153, United States; Sexual Medicine Research Team, Washington, DC 20001, United States; Department of Psychology, Queen's University, Kingston, ON K7L 3N6, Canada; Sexual Medicine Research Team, Washington, DC 20001, United States; Department of Psychology, University of British Columbia, Vancouver, BC V6T 1Z4, Canada; Department of Psychology and Life Sciences, Charles University, Klecany 116 36, Czech Republic; Department of Psychology, Rutgers University, Newark, NJ 08854, United States; San Diego Sexual Medicine, San Diego, CA 92120, United States; Sexual Medicine Research Team, Washington, DC 20001, United States; Department of Urology, Georgetown University Hospital, Washington, DC 20007, United States

**Keywords:** sexual arousal, sexual arousal disorder, tirzepatide, GLP-1 agonists, case report

## Abstract

**Introduction:**

Persistent genital arousal disorder/genitopelvic dysesthesia (PGAD/GPD) is associated with poor quality of life. Due to social stigma and its heterogeneous nature, many patients suffer without treatment.

**Aims:**

This case presents the first example of the successful use of a glucagon-like peptide 1 and glucose-dependent insulinotropic polypeptide receptor agonist (GLP1/GIP RA) medication for the treatment of PGAD/GPD.

**Methods:**

The patient was identified by the Sexual Medicine Research Team, retained as a patient at a sexual medicine clinic, and interviewed for the purposes of this case report.

**Results:**

This case presents a 44-year-old woman with a lifelong history of PGAD/GPD symptoms that caused extreme distress and depression who experienced 95% resolution of her symptoms within 2 days of starting tirzepatide, a GLP1/GIPRA medication, for weight loss.

**Conclusion:**

Increasing benefits of GLP1/GIPRAs are being uncovered, and further studies must investigate the potential for these medications to be used in patients with PGAD/GPD. This study also provides a potential mechanism for decreased arousal resulting from GLP1/GIP receptor activation in attention/reward pathways in the brain.

## Introduction

Persistent genital arousal disorder/genitopelvic dysesthesia (PGAD/GPD) is a disorder characterized by persistent or recurrent unwanted genital arousal that occurs without sexual interest or desire and is associated with distress.^1^^,^^2^ Symptoms are characterized by persistent genital sensations (eg, tingling, buzzing) that do not resolve with orgasm.^1^ PGAD/GPD is estimated to affect 0.6%-4.3% of the population and has been reported in patients as young as 4 years old through patients in their eighties.^2-4^ PGAD/GPD often coexists with mental health difficulties (eg, depression, anxiety, and suicidality) and is associated with a high degree of functional impairment and lower quality of life.^5^^,^^6^ Patients with PGAD/GPD often undergo delays to diagnosis, and the identification of an individual’s precise PGAD/GPD etiology can be challenging due to the multifactorial nature of the condition.^7-9^

We present the case of Ms. M, a 44-year-old woman with a lifelong history of PGAD/GPD symptoms who experienced 95% resolution of her symptoms within 2 days of starting tirzepatide, a GLP1/GIP receptor agonist medication, for weight loss. Ms. M also has a complex psychiatric history, endometriosis status post hysterectomy, Gitelman syndrome, and hypothyroidism.

## Case

During a monthly meeting of the Sexual Medicine Research Team (an international group of medical students, healthcare professionals, researchers, and patient advocates studying sexual medicine), the case was brought to the group’s attention with the patient’s permission. Ms. M was subsequently retained as a patient at a sexual medicine clinic and interviewed separately by two medical students for the purposes of this case report.

Ms. M reported lifelong symptoms of pressure in the wall of the rectum and the vagina combined with perineal spasms, a feeling of fullness in her rectum, and an arousing sensation up to her mid-back, all with left-sided predominance. She also endorsed umbilical hypersensitivity, intermittent weakness and tingling in her legs, and spontaneous nocturnal orgasms. Symptoms were intermittent throughout her life, with periods lasting a week to a month, were triggered by riding in vehicles or stretching, were reproducible with left plantar flexion, and were improved by masturbation until her mid-30s, at which point her symptoms worsened in both temporality and severity. Ms. M became pregnant in her 20s and her symptoms were unchanged by pregnancy and childbirth. She also had multiple laparoscopic surgeries for endometriosis, none of which changed her symptoms. Ms. M’s mental health history includes self-reported major depressive disorder, generalized anxiety disorder, obsessive-compulsive disorder, bipolar disorder, childhood sexual trauma, and a suicide attempt at 10 years old. She has utilized multiple psychiatric medications with no improvement to her PGAD/GPD symptoms; however, she noted that cessation of SNRIs triggered symptoms.

Beginning in 2015, Ms. M began to seek medical assistance for her worsening symptoms, at which point she received an official PGAD/GPD diagnosis and a variety of treatments, including a pudendal nerve block and gluteal muscle dry needling, both of which relieved her symptoms for a few days, as well as pelvic floor physical therapy, topical vaginal gabapentin, and a hysterectomy/salpingectomy due to her history of endometriosis, all of which provided no symptomatic relief. A lumbar spinal MRI was performed which was negative for pathology and physical examinations were unrevealing. By 2020, her symptoms were constant, and Ms. M endorsed increased alcohol intake to cope with the worsening symptoms. Ms. M endorsed feeling suicidal, with retrospective pre-treatment ratings including Persistent Genital Arousal Sensations Questionnaire (PGASQ) score of 58 (out of 60), Pain Catastrophizing Scale (PCS) score of 47 (out of 52), and scores indicative of severe depression and severe anxiety on standardized questionnaires.

Ms. M initially tried tirzepatide injections for weight loss in November of 2022 with 95% relief of PGAD/GPD symptoms two days after her first injection, with continued reproduction of her symptoms on left plantar flexion. Ms. M states that relief from symptoms lasts 7-8 days. After a few months on the medication, she was no longer able to obtain insurance coverage for a prescription of tirzepatide and began taking a compounded peptide tirzepatide from an online distributor, noting that the tirzepatide injections obtained this way felt weaker than her prior prescription injections. After a year of using tirzepatide at a dose of 2.5 mg per injection once a week, Ms. M began to feel that her symptoms were no longer well-controlled and increased her dose to 5 mg per injection once a week with resolution of her symptoms.

At the time of writing, Ms. M’s symptoms are still well-controlled. Ms. M’s current PGASQ score is 4, her PCS score is 4, and she has scores indicative of moderate depression and low anxiety on standardized questionnaires. She has lost 50 lbs and is satisfied with her weight loss. She has experienced side effects that she attributes to the tirzepatide, including nausea, diarrhea, joint pain, and a sunburn-like sensation on her face and neck shortly after injection.

## Discussion

This case report illustrates a newly reported phenomenon: the rapid resolution of lifelong PGAD/GPD symptoms and related severe depression, anxiety, and alcohol use after the administration of tirzepatide. This medication is a glucagon-like peptide-1 (GLP1) and glucose-dependent insulinotropic polypeptide (GIP) receptor agonist medication (GLP1/GIP RA).

The presenting etiology and pathophysiology of PGAD/GPD is highly variable. In 2021, the International Society for the Study of Women’s Sexual Health (ISSWSH) released a consensus statement characterizing the condition according to symptom origin across five regions. This has led to the following diagnostic and treatment algorithm for each region, which is the current standard of care.^7^ Thus, once the contributing factor of the PGAD/GPD symptoms is identified, treatment targets the affected region. End organ dysfunction can include clitoral, vestibular, vulvar, vaginal, and/or urinary tract pathologies.^10–12^ Pelvic or perineal sources of PGAD/GPD symptoms include pelvic floor dysfunction, pelvic congestion syndrome, and pudendal neuropathy.^13-15^ Symptoms may originate from the cauda equina or spinal cord regions.^16^^,^^17^ Finally, conditions in the brain such as epilepsy, psychological factors such as bipolar disorder, and the addition and removal of various psychological medications have all been demonstrated to contribute to PGAD/GPD.^18-23^ While treatment algorithms exist for each region involved, patients may experience symptoms attributable to multiple regions and therefore may undergo extensive and costly medical management with minimal symptomatic relief. Often, the underlying cause of the symptoms is not identified, and patients continue to experience distress, stigma, and shame that may ultimately be life-threatening, as in the case of Ms. M. The current serendipitous observation of the efficacy of tirzepatide against PGAD/GPD provides evidence for an additional form of therapy.

Over the past 10 years, GLP1 RAs have shown benefits in controlling blood glucose levels in people with type II diabetes and promoting weight loss as well as improving cardiovascular function.^24-28^ The more recently developed tirzepatide is a member of the class known as “twincretins,” with GIP RA activity potentiating the effects of the GLP RA and resulting in comparable, if not better, control of diabetes and weight.^29-33^ More recently, GLP1 and GLP1/GIP RAs have been proposed to reduce addictive behaviors to various substances, including alcohol, nicotine, opioids, and stimulants, primarily in animal models but with some efficacy shown for reduction of alcohol use in humans as well.^34-43^ The proposed mechanism for this effect is related to tirzepatide’s ability to control appetitive desires. The brain processes reward via dopaminergic signaling as part of the mesolimbic and mesocortical attention/reward pathway, beginning in neurons in the ventral tegmental area that communicate with the prefrontal cortex, basolateral amygdala, hippocampus, and nucleus accumbens.^44^ Dopaminergic neurons fire either tonically, providing a background level of dopaminergic signaling, or phasically, activating in short, strong bursts that activate low-affinity receptors and provide stronger reward responses.^45^ GLP1 and GIP receptors have been found throughout the brain and, notably, within brain regions that are involved in reward signaling.^46-51^ These receptors are also found on neurons in the medial preoptic area (mPOA) that project to the dorsomedial hypothalamus to reduce food intake.^52^ Although the exact mechanism for GLP1 RA and dopaminergic signaling is unknown, it is possible that GLP1 RAs increase tonic dopaminergic firing without increasing phasic dopaminergic firing, which may result in a blunting of the phasic bursts that are responsible for the pleasurable experiences associated with the anticipation of drug use, hence leading to the addiction- and appetite-suppressing effects of tirzepatide.^53^ Dopamine release in the mPOA follows a similar pattern to that in the mesolimbic/mesocortical system, and mPOA dopamine release acting on D1 receptors is important for the regulation of genital blood flow. The ability of phasic release in the mPOA to be taken over by drugs that induce slight tonic release (eg, the nicotinic cholinergic agonist varenicline and dopamine agonist pramipexol), or that inhibit release (the selective benzodiazepine 1 receptor agonist zolpidem) or block dopamine receptors (eg, atypical antipsychotic medications like olanzapine, brexpiprazole, and paliperidone) may underlie the amelioration of PGAD/GPD symptoms, as those drugs all have shown efficacy relieving feelings of genital engorgement in case reports.^54–58^

Thus, the effects of GLP1 RAs on Ms. M’s PGAD/GPD symptoms may mimic the proposed mechanism for GLP1 RAs and addiction.^59–61^ Regardless of tirzepatide’s mechanism of action for Ms. M, this case presents a novel pharmacological treatment option for a patient with a lifelong history of undifferentiated PGAD/GPD. Another GLP1 RA, semaglutide, was recently reported to contribute to a reduction in PGAD/GPD symptoms when combined with additional medications over a longer period of time.^62^ The present case provides plausible mechanisms for GLP1/GIP RAs in patients experiencing PGAD/GPD. Future studies are needed to explore the efficacy of this treatment option in PGAD/GPD patients and to elucidate more fully its mechanism of action.
